# Protein cargo of Nef-containing exosomal extracellular vesicles may predict HIV-associated Neurocognitive Impairment status

**DOI:** 10.21203/rs.3.rs-2740135/v2

**Published:** 2023-06-23

**Authors:** Allen Caobi, Rachel Werne, Mario Gomez, Mickensone Andre, Charo Thomas, Adriana Yndart, Francisco Lima-Hernandez, Madhavan Nair, Andrea D Raymond

**Affiliations:** 1 Herbert Wertheim College of Medicine at Florida International University, Department of Immunology and Nanomedicine, Miami, 33199, USA; 2 College of Arts, Sciences, and Education at Florida International University; Department of Chemistry and Biochemistry; Advanced Mass Spectrometry Facility, Miami, FL 33199

## Abstract

Exosomal extracellular vesicles (xEVs) in plasma and cerebrospinal fluid (CSF) of aviremic people living with HIV/AIDS (PLWHA) contain the HIV Negative factor (Nef) protein. However, the role of xEVs and Nef-containing-xEVs(xEV-Nef) in HIV-associated neuropathology is unknown. Here we performed a cross-sectional analysis of the content of xEVs derived from matched serum and CSF samples of PLWHAs diagnosed with either asymptomatic neurocognitive impairment (ANI), mild neurocognitive disorder (MND), or HIV-associated dementia (HAD). The overall objective was to determine whether the content of the matched xEVs derived plasma or CSF correlated with the neurocognitive impairment (NCI) status. The size and protein content of the xEVs were characterized via dynamic light scattering (DLS) and LC-MS/MS, respectively. xEV size was not significantly different between ANI, MND, or HAD groups. CSF of PLWHAs with NCI contained significantly more xEVs than matched plasma. xEV-Nef CSF concentration was elevated in PLWHAs with NCI and correlated with CD4 T-cell count. Plasma-derived xEV protein profiles from PLWHAs with ANI or MND differed from PLWHAs without NCI. Over-representation analysis using Reactome and KEGG databases show proteins involved in pathways associated with heme scavenging, signaling(MAP kinase and integrin-alpha),Toll-like receptor regulation, clot formation, complement, and cytosolic calcium level were elevated in MND. Pathways upregulated within the ANI group involved high-density lipid (HDL) remodeling, post-translational protein phosphorylation, and platelet activation. Overall, the data shows that xEV protein profiles of ANI and MND differ, suggesting protein profiles of peripheral xEVs, xEV-Nef, and CD4 T-cell count may discern NCI status.

## Introduction

Despite successful suppression of HIV viral loads by anti-retroviral therapy (ART) to undetectable levels, many aviremic HIV-infected individuals still develop neurocognitive deficits[1-6]. The exact mechanism(s) of HIV-associated neurocognitive disorder (HAND) in aviremic patients is still unclear. HAND ranges from asymptomatic neurocognitive impairment (ANI) to mild neurocognitive disorder (MND),with concentration and memory issues, to HIV-associated dementia (HAD)[6, 7]. Several degrees of HAND were defined, with detailed standards, in 1991 by the American Academy of Neurology (AAN) [8]. These standards described two degrees of cognitive impairment, minor cognitive motor disorder (MCMD), a less severe form of behavioral and cognitive motor impairment, and HIV-associated dementia (HAD), which was more severe. However, to categorize the neurocognitive impairments in a post-HAART era, a revised classification was proposed in 2007, which now included milder forms of NCI [9]. Most importantly, this revision removed the possibility of diagnosing HIV NCI based on noncognitive and neuromotor psychiatric changes, which include alterations in mood and personality. Three syndromes, in the context of HAND, were established and defined with more precise benchmarks, ANI, MND, and HAD. Both ANI and MND were proposed to better characterize the neurocognitive deficits seen within milder stages of HAND, thereby replacing the term MCMD. The development of these new criteria is detailed in A. Antinori, G. Arendt, J. T. Becker, et al [9]. Mechanisms of NCI have been associated with neuroinflammation, immune activation, and signaling cascade dysregulation[10-13]. At the time the CSF and plasma samples were obtained from patients, the newer and more precise benchmarks for NCI have yet to be developed and implemented. As such, samples classified as MCMD, will be reclassified as MND, in this study. HIV-associated NCI is assessed via a battery of neurological psychiatric tests -Trail making tests A, trail making test B, and DSY as defined by Frascati criteria[9, 14]. However, these tests are not only time-consuming and objective, but have been argued to generate an elevated percentage of false positives since the Frascati criteria are too liberal[15]. Early studies have demonstrated that in the era of highly active antiretroviral therapy (HAART), neuronal injury, inflammation, and cognitive impairment persist[16]. This suggest that other factors not directly related to HIV infection may play a role in HAND. Currently, there are no non-invasive therapeutic methods or monitoring systems that directly address the neurocognitive impairment (NCI) in aviremic HIV+ subjects. Recent studies have attempted to optimize the neurocognitive criteria employed in the detection of HIV-associated brain abnormalities, including employing neuroimaging tools to assess metabolite and structural brain imaging markers in PLWH [17]. Individuals defined as neurocognitively impaired by Frascati criteria exhibited less cortical gray matter, larger sulcal cerebrospinal fluid(CSF) volumes, and more evidence of neuroinflammation[17]. There is now evidence that extracellular vesicles(EVs) and other factors in the plasma may play a role in HIV neurocognitive disorder[18, 19]. We theorize that the EVs released from neuronal and immune cells within the central nervous system and periphery may play a role in neurocognitive impairment (NCI) of aviremic HIV-infected patients.

Of the three classes of EVs – exosomes, microvesicles, and apoptotic bodies, exosomal extracellular vesicles (xEVs) are the smallest ranging in size from 30-150 nm vesicles and are derived from the plasma membrane, thus capable of invagination and encapsulation of cell material such as proteins, RNA, and DNA[20-22]. These vesicles can then fuse with the plasma membrane and be subsequently released into the extracellular space, internalized in a juxtracrine, paracrine, or endocrine manner, thus transferring its content to a recipient cell. Once internalized, the exosomal content can alter the recipient cell’s molecular profile by various mechanisms (alterations in transcription, translation, protein modification, signaling cascades, etc.). The xEVs are hypothesized to play a large role in cell-to-cell communication[23]. xEVs were initially identified in cells of hematopoietic lineage but have since been identified in a variety of cells including dendritic cells[24], neurons[25, 26], tumor cells[27, 28], and epithelial cells[29, 30]. xEVs have been observed in body fluids such as blood, urine, and cerebrospinal fluid, easily cross cellular barriers (i.e., blood-brain-barrier (BBB), and may play a role clinical diagnosis[31-33].

The rationale for this study is based on the observation that HIV induces the release of xEVs from infected cells for viral dissemination and pathogenesis. HIV-accessory proteins, such as the negative factor (Nef), have been identified in the xEVs[34, 35], indicating that xEVs secreted from HIV-infected cells transport viral components throughout host fluids and tissues. Viral proteins such as HIV Nef, a highly expressed accessory protein promotes viral replication, disrupts host immunity by rerouting cell-surface proteins, and counteracts host immune defenses by employing the secretory or endocytic pathways to degrade or sequester its targets [36, 37]. HIV+ individuals (viremic and aviremic) possess higher concentrations of xEVs than non-infected individuals[18, 38]. Nef-containing EVs function to promote EV secretion, increased MVBs within cells, and induces apoptosis within CD4+ T-cells [39-42]. Given that Nef has several functions, Nef-EVs could potentially promote decay of CD4+ T-cell populations, promote CD8+ T-cell activity, CXCR4-mediated apoptosis, and ADAM17 activation increasing CD4+ T-cell permissiveness to HIV-1 [43-47]. Exosomal and cellular miRNA profiles are modulated by the HIV-1 Nef protein, affecting both HIV-1 pathogenesis and viral replication modulated by host RNAi [48]. This suggests that viral dissemination and pathogenesis via xEV transport may be involved in the development of HAND in aviremic patients. It is estimated that 50-60% of the HIV+ population is affected by HAND[49]. Therefore, methods to easily monitor, diagnose, and treat HAND are needed. The mechanism of HAND in aviremic patients is still unclear and currently there are no therapeutic methods or monitoring systems for neurocognitive impairment in aviremic HIV+ subjects. Theoretically, xEVs released in peripheral blood and/or the cerebrospinal fluid (CSF) contribute to the development of HAND in aviremic HIV+ subjects and that the xEV content could serve as an indicator of HAND.

To date, no studies have examined the association of xEVs and Nef in HIV neuropathogenesis in aviremic PLWHAs. Here we present preliminary findings of a cross-sectional study that evaluates whether xEVs and Nef-containing-xEVs detected in plasma and CSF correlate with the neurocognitive status of aviremic PLWHAs. This preliminary study showed that NCI status correlated with the xEV cargo, concentration, and xEV-Nef levels. Peripheral exosomal protein profiles of aviremic HIV+ donors with ANI, MND, or HAD differed from those aviremic HIV+ donors with no NCI suggesting that xEV cargo could identify NCI status. Direct comparison of matched plasma and CSF xEV proteins showed that plasma-derived xEVs, protein expression profile differed between NCI status. Nef-xEVs were contained at higher levels in the CSF of donors with NCI and positively correlated with the total CD4-lymphocyte count(TCL). Lastly, a linear regression of the TCL and xEV Nef concentration could predict NCI status. This study is the first to compare xEV in plasma and CSF and provides evidence that xEV protein cargo may indicate NCI status in PLWHAs.

## Results

Cryopreserved matched plasma and CSF acquired from NNTC were used to determine whether the protein content of xEVs and Nef-containing-xEVs correlate with the neurocognitive impairment(NCI) status of PLWHAs.Since most of the study population described in Table 1 had no significant neurocognitive impairment(44%). The study population primarily male subset was comprised of ten individuals who were primarily MSMs ranging from age 30-69 years-old, 17% were Black, 31%, Hispanic, and 72% White. Most of the cohort(86%) did not use IV drugs and 50% of participants presented with some degree of neurocognitive impairment, as denoted in Table 1.

### Charcterization of EVs in HIV-associated NCI

**Figure 1.**
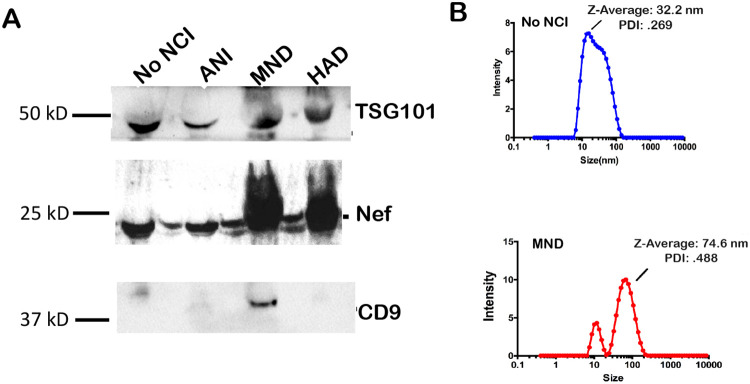
Exosome Characterization. xEV characterization and size (A)Immunoblot of representative exosomal lysates for markers Alix (96 kDa) and CD9 (25 kDa). SDS/PAGE and western blot (immunoblot) analysis performed using exosomal lysates (10 ug total protein). Data show C9 levels reduced in MND. (B). Size determination of plasma exosomal preparations via dynamic light scattering (DLS) (Zetasizer, Malvern.com

Exosomes were isolated via differential ultracentrifugation (UC) and exosomal markers were detected via western blot. Exosome size was ascertained via dynamic light scattering (DLS) using the Malvern Zetasizer Nano. Interestingly, the exosomal marker TSG101 was expressed in the EVs- derived from all participants with no NCI or NCI( ANI, MND and HAD) while CD9, a tetraspanin was only found in MND(Figure 1A, bottom panel ). The HIV Nef protein was detected in all groups. However, Nef appears to be expressed higher in exosomes of patients with MND or HAD (Figure 1A, middle panel). Additionally, isolated exosomes were also characterized by their size. Both individuals with and without neurocognitive impairment presented with EVs within the expected size range of 30-150nm in diameter (Figure 1B). However, the average EV size was found to be greater in individuals with NCI.

**Figure 2.**
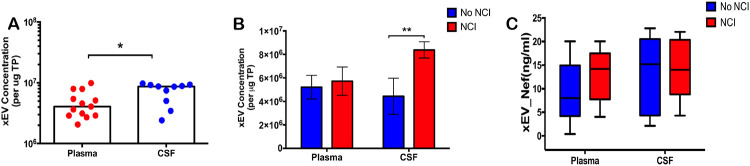
Exosomal EVs containing Nef are elevated in CSF of PLWHAs with neurocognitive impairment. (A) Total xEVs isolated from plasma (n=13) or CSF (n=10) using ExoquickTM followed by Ultra-centrifugation were enumerated via Exocet ELISA (System Biosciences). (B) xEVs from the plasma (n=13) and CSF (n=9) of PLWHAs with NCI compared to donors that did not have NCI Samples form plasma and CSF of PLWHAs (C) Nef-levels in plasma- and CSF-derived xEVs from normal or NCI PLWHAs was measured via anti-Nef ELISA. Statistical significance determined using Unpaired t-Test or Two-Way ANONA; post-hoc analysis – Sidak’s test. *p<.05, **p<.01, and ns=not significant.

To determine whether there is a variance in the concentration of xEVs and/or Nef-containing-xEVs, in plasma or CSF; ex-vivo data using plasma from ACTG was collected via an Exocet ELISA. Total EVs isolated from patient plasma and CSF were tallied and compared. A significantly greater concentration of EVs was present within patient CSF, relative to patient plasma (Figure 2A). An elevated concentration of EVs was detected in CSF in patients presenting with NCI, relative to asymptomatic HIV-infected patients (Figure 2B). To ascertain if Nef-containing EVs are predominant within the EV population in PLWHA presenting with NCI, the number of EVs containing Nef was attained via an anti-Nef ELISA. There was no significant difference between individuals with NCI versus those without, within patient CSF (Figure 2C). However, there was a trend of increasing Nef-containing EVs within the plasma of NCI presenting patients. This data suggests that EV concentration may function as a biomarker for NCI, as a greater presence of EVs may be found within the CSF of PLWHA presenting with NCI.

**Figure 3.**
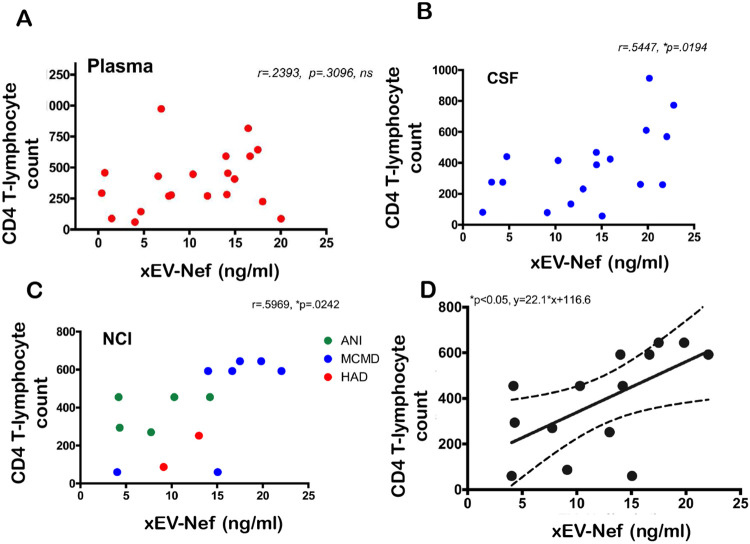
xEV-Nef level correlates with CD T-cell count xEV-Nef concentration in (A) plasma (n=20, XY pairs) and (B) CSF (n=19, XY pairs) was correlated with TLCCD4-C independent of compartment. (C)Correlation of xEV and CD4-C in PLWHAs with NCI. Statistical significance determined using parametric analysis Pearson, r and *p<.05.

Nef-contained EVs did not correlate with HIV-induced NCI status and CD4 T-cell count, the Total Lymphocyte Count of CD4+ T-cells (TLC-CD4)(Figure 3A). No correlation was found between xEV-Nef concentration and TLC-CD4 in plasma (Figure 3A); However, a positive correlation was observed in patient CSF (Figure 3B) and when grouped by NCI (Figure 3C). Linear regression analysis clearly demonstrates that the T-lymphocyte count and xEV-Nef concentration in the CSF can be used to predict NCI status(Figure 3D). This data suggests that Nef-EVs may have a role in NCI development in PLWHA.

**Figure 4:**
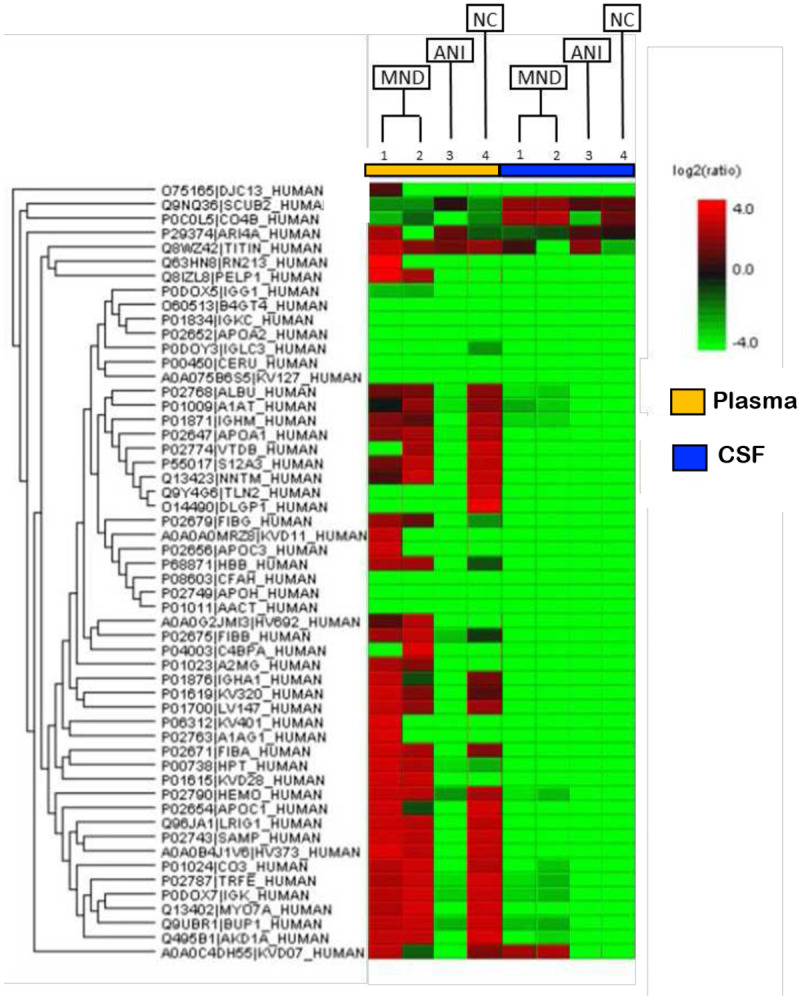
Biomarker of NCI in xEV composition of PLWHAs. Protein content of xEVs isolated from plasma and matched CSF sample were analyzed via mass spectrometry (LC-MS/MS). Samples of either ANI or MND NCI were compared to non-NCI control (NC). Depicted here is the protein content of xEVs from four HIV+ patients, patients 1 and 2 present with MND and are HIV+. Patient 3 presents with HIV+ with ANI. Patient 4 is HIV+ patient without NCI. (Cell color represents the log2(ratio) to the average area across different samples)

We next compare the protein content of plasma- and CSF-derived EV protein content via mass spectrometry (LC-MS/MS). The data indicates that protein expression profiles varied dependent on NCI status(Figure 4). Very little protein was detected in CSF-derived xEV via LC-MS/MS differences observed are from plasma-derived samples suggesting that CSF-derived xEV do not contain a great deal of protein or our instrument was simply not sensitive enough to detect sufficient protein in these samples. as there is no significant difference in protein content between NCI groups in CSF-derived EVs. However, these preliminary findings suggest that differences in protein expression may be exploited to generate an exosomal protein profile, that may serve as a biomarker of NCI status.

To further assess whether the protein profile observed could be linked to NCI in PLWHs we closely examined the proteins differentially expressed in the NCI and ANI donors. The proteins observed to be upregulated relative to the control, for both MND patients in plasma, are: PELP1, FGG, HBB, IGHV1-69-2, FGB, A2M, HP, and IGKV2D-28 (Table 3). However, only two proteins were found to be differentially downregulated, relative to the NC, for the MND patients: TLN2 and DLGAP1 (Table 3). There were no proteins found to be significantly upregulated in the ANI group relative to the NC. However, multiple proteins were found to be downregulated in the ANI group, relative to the NC: ALB, SERPINA1, IGHM, APOA1, GC, SLC12A3, NNT, TLN2, DLGAP1, IGHA1, IGKV3-20, IGLV1-47, FGA, APOC1, LRIG1, APCS, IGHV3-73, C3, TF IGKC, MYO7A, UPB1, ANKDD1A, and IGKV3D-7 (Table 3).

**Figure 5.**
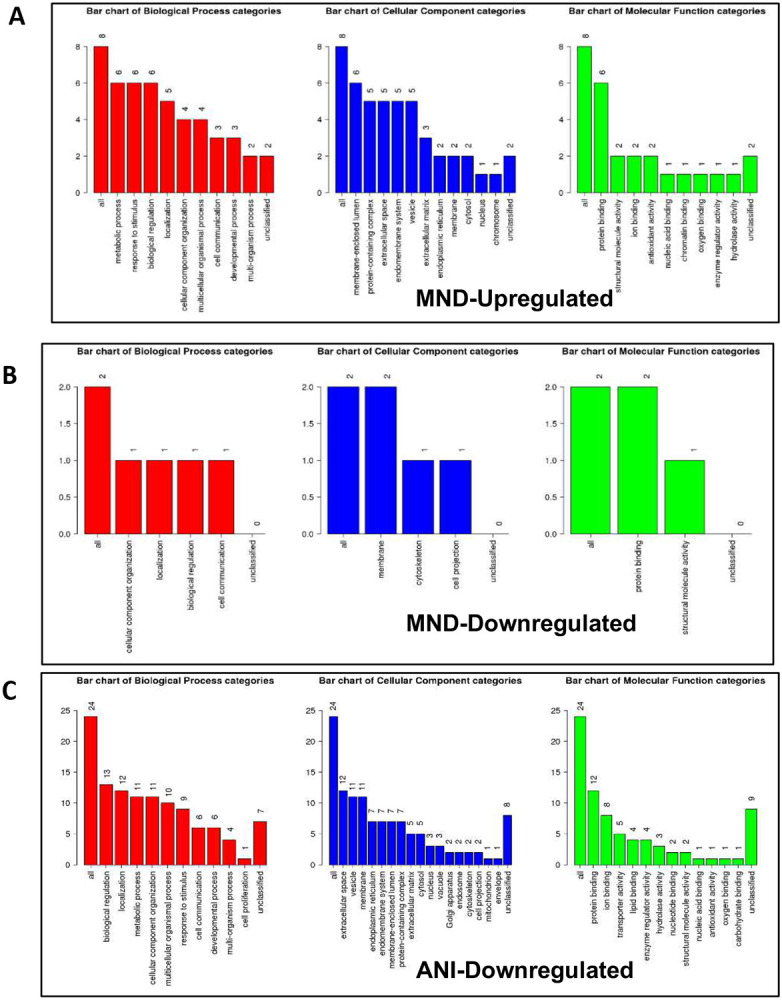
HIV induces differential gene expression in exosomes, varying by NCI status. EV-derived genes involved in key biological processes, cellular components, and molecular functions. (A) A GO Slim classification of the up or downregulated proteins within each subset ratio of HIV-infected patient plasma vs Uninfected patient plasma, providing a high-level functional classification of the significant differentially expressed genes. GO Slim was analyzed and acquired via the WEB-based GEne SeT AnaLysis Toolkit (Webgestalt).

To develop a profile of EV-bound proteins correlating with NCI status, A GO Slim classification of the up or downregulated proteins was generated. Patients presenting with MND had a higher concentration of EV-bound proteins involved in biological processes including response to stimulus, biological regulation, localization, and metabolic processes (Figure 5). MND patient samples also presented with a downregulation of proteins primarily involved in cellular component organization, within the biological process category (Figure 5). ANI samples demonstrated a similar set of modulated biological processes, including biological regulation, metabolic process, and localization among the top 4 biological processes categories for both groups (Figure 5). Of note, ANI samples had at least twice as many proteins involved in the extracellular space, the vesicle, membrane, and transporter activity (Figure 5).

**Figure 6.**
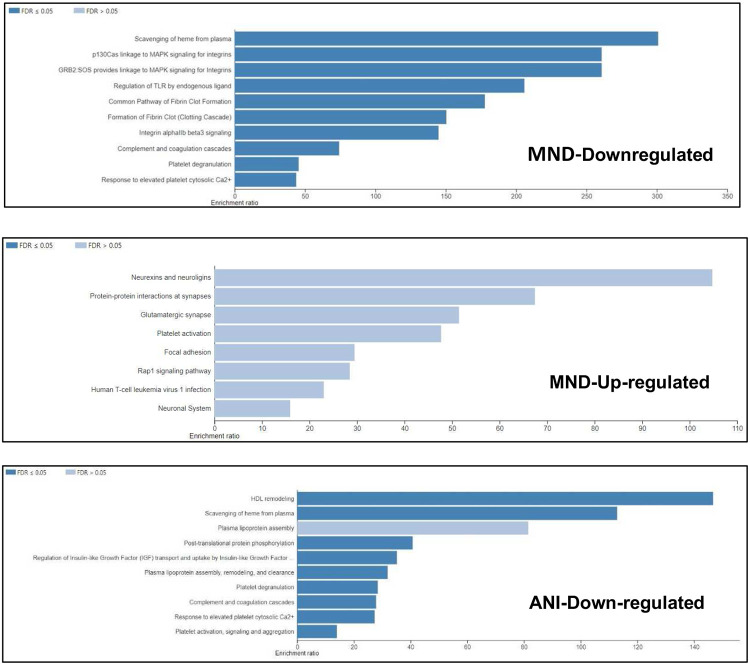
HIV induces differential gene expression, varying by NCI status, modulating various pathways critical to the immune system, the CNS, and gas-exchange-associated pathways. (A) Bar charts depicting modulated pathways because of HIV-induced NCI, relative to NC. Data was analyzed via the WebGestalt online tool using a complementary method for enrichment analysis, over-representation analysis (ORA), with the Reactome and KEGG functional database for pathway analysis.

An over-representation analysis (ORA) using the Reactome and KEGG functional database for pathway analyses was performed to determine which pathways are affected by these modulated proteins. The proteins upregulated within the MND samples are involved in the scavenging of heme from plasma, MAPK signaling, TLR regulation, fibrin clot formation, integrin alpha beta 3 signaling, complement and coagulation cascades, platelet degranulation and response to an elevated platelet cytosolic calcium level (Figure 6). In contrast, the downregulated proteins, within the MND samples, are much more involved in the CNS, involved in pathways such as: neurexins and neuroligins, protein-protein interactions at synapses, the glutamatergic synapse, and the neuronal system (Figure 6). Of note, ANI samples-derived protein modulates some pathways also modulated by MND’s upregulated proteins, such as: complement and coagulation cascades, platelet degranulation, and scavenging of heme from plasma (Figure 6). However, a few differences may be observed between the pathways modulated by ANI and MND’s upregulated proteins, such as: HDL remodeling, post-translational protein phosphorylation, platelet activation, signaling and aggregation (Figure 6).

Overall, results show that both CSF-and plasma-derived exosomes from HIV-infected individuals contained Nef-xEVs which when coupled with their total CD4 T-cell counts significantly correlated with NCI status suggesting that exosomal EVs containing the HIV Nef protein may be used as a relatively non-invasive predictive biomarker of NCI status in aviremic PLWHAs.

## Discussion

Infection with HIV may result in neurological complications. Identifying the degree of severity of an HIV-associated neurological complication is critical in determining the optimal treatment for the patient. The neuropsychiatric tests used to assess neurocognitive impairment can be cumbersome and somewhat subjective. Thus, in this study, we aimed to provide an alternative method to evaluate NCI status in PLWHA in a manner that is not as subjective as the battery of neuropsychological examinations but instead quantifiable. Recent studies have assessed other non-invasive methods of ascertaining NCI status, such as examining metabolic risk factors and multimodal magnetic resonance imaging data via machine learning [45, 46]. However, both options still required clinical observations. The data demonstrates that it is possible to ascertain patient NCI status qualitatively by analyzing patient protein and miRNA exosome profiles. Profiles could be used as supportive evidence of NCI status. Exosomes characterized from patient plasma were found to be within the expected size range for exosomes, at 32.2 nm and 74.6 nm in Z-Average, for patients without NCI and patients presenting with MND, respectively (Figure 1). Additionally, the exosomal marker, TSG101, was found to be present in both control and NCI samples (Figure 1). However, there was a notable absence of CD9 expression, a standard exosomal biomarker in EVs derived patient plasma with MND (Figure 1). This data confirms that the isolated EVs were composed primarily of exosomes amd perhaps other EVs(i.e. microvesicles). EV concentration with respect to NCI status was investigated, and exosomal EVs containing Nef were elevated in the CSF of PLWHAs with NCI (Figure 2). Also, exosome concentration was elevated in the CSF of individuals presenting with NCI (Figure 3a-b). This data implies a relationship between EV-bound Nef and patient neurocognitive status. Nef is transported via EVs to recipient cells [47]. This EV-bound secreted Nef is also known to enhance its export from infected cells by increasing exosome production [48]. Studies show that Nef-EVs also initiate activation-induced apoptosis in resting CD4+ T- lymphocytes suggesting that it may be involved in the notable loss of CD4+ T-cells observed in AIDS [48]. EV-bound Nef rearranges lipid rafts by re-localizing TREM1, and TLR4, an amplifier of the innate immune response and a critical inflammatory receptor, respectively. Cholesterol metabolism is also modulated by EV-Nef, ultimately resulting in Nef-EVs potentiating the inflammatory response [49].

Although other HIV proteins such as Tat and GP120 are found in the peripheral blood and can contribute to HIV neuropathogenesis. Tat has not been detected directly associated with EVs. Tat has been shown to up-regulate the release of specific microRNAs(miRs), miR-7 and miR-9 in exosomes released by astrocytes [50, 51]. EVs released from HIV infected cells were shown to carry the HIV Env(gp120) which facilitated HIV infection[52]. Although, these reports have shown that other HIV proteins can be found in EVs, we focused on Nef due to its multifactorial functions and cellular locations. Nef has been shown to be neurotoxic, disrupts signaling cascades in immune cells, lowers surface expression of CD4 and MHC-class I, and promotes HIV dissemination[53, 54]. Since Nef can be found on the cell surface in lipid rafts and in the cytosol, Nef is likely to be detected in both exosomal and microvesicular EVs. These Nef-containing EVs are likely to have multiple effect on cells.

Our data shows that EV-Nef concentration in the CSF correlates with CD4+ T-Cell count (Figure 4b). Additionally, EV-Nef Concentration correlated positively with NCI status in PLWHA (Figure 4c). Altogether, this data strongly suggests a potential link between EV-bound Nef and NCI in PLWHA that demonstrates the capacity of EVs derived from PLWHA to be biomarkers of NCI status(Figure 5). Several differentially expressed proteins were also present within EVs derived from PLWHA that were dependent on NCI status (Figure 4). These proteins may be stratified and separated into groups detailing their expression relative to a non-NCI control (Table 2). Based on this data, these proteins, when released in exosomes, may serve as biomarkers of MND or ANI.

Furthermore, a profile of EV-bound protein dependent on NCI status was generated based on biological processes, cellular components, and molecular functions of the protein mentioned above groups (Figure 6). Individuals presenting with ANI could be more easily classified as they presented with at least twice as many proteins involved in the extracellular space, the vesicle, membrane, and transporter activity (Figure 6). On the other hand, MND may be more easily profiled by ascertaining which pathways were modulated (Figure 6). MND samples displayed a more significant modulation in CNS-associated pathways. These pathways include neurexins and neuroligins, protein-protein interactions at synapses, the glutamatergic synapse, and the neuronal system (Figure 6). These pathways are critical to neuronal cell function This correlates with the understanding of the characteristics of HAND which include neuronal cell death in specific subcortical brain regions such as the basal ganglia and hippocampus as well as the thinning of the cerebral cortex [55-57]. Approximately 55-65% of PLWHA and undergoing ART have been reported to experience HIV-related fatigue (HRF) [58, 59]. A study performed on patients treated with highly active antiretroviral therapy (HAART) found that apolipoprotein B (ApoB) has a negative correlation with fatigue severity [60]. However, in naïve HIV-infected patients, ApoA1 instead presents with a positive correlation with fatigue severity [60]. In our study ApoB was downregulated. Significantly less proteins in the CSF-derived EV samples, unlike other groups such as Guha et al., who found common proteins, inflammation markers, and markers related to cells of the CNS [61]. The reason for the decreased protein concentration from the CSF samples may be due to differential technique performance, technical difficulties, or sensitivity of the LC-MS/MS. Levels of A2M in serum-exosomes of alcohol consumers have been demonstrated to be lower than those in healthy individuals suggesting that alcohol consumers may be at an increased risk of neurological impairments [62]. Additionally, a study by Varma et al., suggests that alpha-2-macroglobulin (A2M) may play a role in neurocognitive dysfunction as patients with an increased susceptibility to Alzheimer’s disease presented with an elevated level of A2M in serum [63]. Similarly, in this study exosomes derived from MND patients presented with an elevated level of A2M as well. Both studies suggest that A2M may be critical in neurocognitive dysfunction.

While the initial findings of this study are exciting, there were some limitations of the study. First, the exosomal protein profiles identified via mass spectrometry used only cryopreserved plasma and CSF. However, it is unknown whether cryopreservation damages or alters EV contents. In our subsequent study, we will perform a comparative analysis of EV cargo in cryopreserved vs. fresh plasma and CSF. Secondly, the sample size of PLWHAs with NCI used was small. However, our objective was to determine whether the exosomal protein profiles differed significantly between CSF and plasma and whether these difference may predict NCI status. Preliminary findings suggest that the profiles differ and that exosomal EV cargo can differentiate between MND and ANI. Lastly, we could not assess whether sex-based differences in exosome cargo occur because the specimens were all from male PLWHAs. We intend to expand the study shortly and will address these limitations. Overall, we show that exosome protein cargo, including the Nef protein, may indicate and predict NCI status in aviremic PLWHAs.

## Methods

### Study Subjects and Ethics Statement

The specimens used in this study were selected from banked plasma and cerebrospinal fluid(CSF) specimens in the National NeuroAIDS Tissue Consortium (NNTC). The NNTC studies were conducted in accordance with human subject protection protocols at participating institutions. Written informed consents were obtained for subjects at each collection sites in the United States. Samples were received from sites in Texas and New York. All experimental protocols were approved by Institutional review boards (IRBs) of respective sites. These IRBs managed the protocols inolved in the protection of human subjects: 1) The University of Texas Medical Branch Office of Research Subject Protections and 2) Mount Sinai Medical Center Program for the Protection of Human Subjects. Neurocognitive assessment scores were provided by NNTC, Frascati indexes were used for this study

#### Study Design.

Clinical specimens were acquired from the National NeuroAIDS Tissue Consortium (NNTC) as part of request R489. All studies adhered to the ethical guidelines of the National Institutes of Health and the Florida International University, Miami Florida institutional review Board. NNTC provided neuropsychological diagnosis based on Frascati guidelines [9, 14]. Cryopreserved matched plasma and CSF specimens were acquired from thirty-six participants. Protein cargo of exosomes derived from the CSF and/or plasma of the patients diagnosed with HAND - ANI, MND, or HAD have been identified via mass spectrometry and compared.

#### Study Population.

Specimens collected from demographically matched PLWHs without neurocognitive impairment and PLWHs diagnosed with either ANI, MND, or HAD(Table 1). Cryopreserved specimens were received National Neuro AIDS Tissue Consortium. Table 1 shows that the cohort consisted primarily of males, specifically, men-who-have-sex-with-men(MSMs) collected from NNTC sites - Manhattan Brain Bank and from the BRI at Texas State University. The population was 17% Black, 11% Hispanic, and 72% White, non-Hispanics. Overall, the specimens collected were from PLWHs with (44%) and without NCI(44%).

#### Nef Enzyme Linked Immunosorbent Assay (ELISA).

Nef concentration was measured using a commercially available anti-Nef ELISA kit (Immunodx.com, Woburn, MA). Briefly, the Nef reference was diluted 1:1 with 150 μl of diluent buffer and then 3x-serial dilution performed. Exosomes lysates were diluted 1:2 in component C and placed on the 96 well plate coated with anti-Nef monoclonal antibody. The plate was covered and incubated at room temperature (RT) for 1 hour. The plate was emptied and washed three times with wash buffer (Component B), and then patted dry. Detector reagent (Component E), 100 μl was added to each well and the plate incubated at RT for 1 hour in the dark. Plates were washed and tapped dry as previously described. The TMB substrate (Component F) was added to each well and blue color developed within 5-10 minutes. The reaction was stopped by adding 50 μl of Stop solution (Component G). After reaction stopped the color changed to yellow and was read at 450 nm on spectrometer (Biotek).

#### Extracellular Vesicles (EV)/Exosome Isolation.

EVs were isolated from matched cryopreserved plasma and CSF samples using standard differential centrifugation as described by Raposo et al.[22]. Briefly, 500 μl of plasma was precleared by centrifuging the sample at 3000g for 10 minutes to pellet any cells or debris, supernatants were centrifuged at 2000 g for 20 minutes, followed by 10,000 g for 20 minutes and then final centrifuge at 100,000 g for 70 minutes. EV Pellet was resuspended in PBS (250 μl) and then filtered (0.2 μM). EV were either used immediately or stored at −80 °C prior to use.

#### Liquid Chromatography-Tandem Mass Spectrometry (LC-MS/MS).

Proteins from exosomal lysates were prepared for LC-MS/MS by using in-solution digest (ThermoScientific.com). LC-MS/MS analysis was conducted on a Brunker Tims-TOF instrument operated in positive (+) ion mode in the mass range from 300 – 2100. Chromatography separation was conducting utilizing a 46-minute-long LC method with Optima grade water (0.1% Formic acid) as the aqueous phase and Optima Grade Acetonitrile with 0.1 % Formic as the organic phase. The in-solution digested extracts were diluted 1:5 in 50:50 MeOH:Water (0.1% formic acid) and stored in siliconized glass inserts placed inside a sampling vial and then loaded on to a Shimadzu Prominence HPLC autosampler. Thereafter, 20 μl aliquots were loaded into the HPLC for separation prior to MS analysis. Mass spectrometry was calibrated with Tune Mixture (Agilent.com) utilizing 6 calibration points between 322 and 2121 with a reported standard deviation <1 ppm. Peptide fragmentation was conducted with Collision Induced Dissociation of the 25 most abundant precursors. Post data acquisition, the raw mass spectrometry data was processed with Peaks Studio analysis suite. SPIDER module within PEAKS were used to identify proteins. False discovery rate(FDR) of 1%, a minimum score of 30 for modified peptides, and tandem mass spectrometry searches were conducted with a 0.5 Da tolerance. Data comparing protein expression across NCI status presented as heatmaps.

#### Statistical Analysis and Bioinformatics:

Statistical significance (p-value <0.05) was determined via One- and two-way ANOVAs with post-hoc test (Kruskal-Wallis test) or (Tukey's multiple comparisons test). Linear regression was performed using GraphPad Prism 9. Data was graphed and analyzed using statistical package within GraphPad Prism 9. Heat map proteins that were significantly modulated were chosen and then grouped by expression level (up/downregulated). Genecards.org was utilized to generated gene sets using the UniProtKB/Swiss-Prot identification numbers provided within the heat map. These gene sets, for the MND/ANI NCI status groups, were analyzed using bioinformatics analysis. Bioinformatic analysis was performed using the functional enrichment analysis web tool Web-based Gene SeT AnaLysis Toolkit (WebGestalt) to perform Over-Representation Analysis (ORA), determining the Gene Ontology (GO) categories with significantly enriched gene numbers [64]. The enriched gene set Kyoto Encyclopedia of Genes and Genomes (KEGG) or reactome pathway module was employed via the WebGestalt software.

## Figures and Tables

**Table 1.** Demographics and neurological clinical diagnosis

Demographics and Characteristics of Study Population(n=36)	n(%)
**Age**	
30-39	4 (11)
40-49	17 (47)
50-59	11 (31)
60-69	4 (11)
**Race**	
Black	6 (17)
White	26 (72)
Other	4 (11)
**Ethnicity**	
Hispanic/Latino	11 (31)
Not Hispanic/Latino	25 (69)
**IV Drug use**	
Yes	5 (14)
No	31 (86)
**Men who have sex with men(MSM)**	
Yes	29 (81)
No	7 (19)
**Clinical Diagnosis**	
• No significant impairment on NP testing	16 (44)
• Neurological impairment – does not meet criteria for syndromic disorder	2 (6)
• Asymptomatic Neurocognitive Impairment (ANI)	5 (14)
• Mild Neurocognitive Disorder (MND)	7 (19)
• HIV-Associated Dementia (HAD)	4 (11)
• Neuropsychological impairment or dementia due to other cause	1 (3)
• Unknown	1 (3)

**Table 3.** List of proteins differentially expressed in or on EVs derived from patient plasma. The proteins listed here are listed using the most up-to-date gene symbol, according to GeneCards, The Human Gene Database (genecards.org). The proteins listed in Figure 4 were compared groupwise, by NCI status against the NC. On the left column, are listed proteins which were found to be upregulated in both MND patients versus NC. In the middle column, are listed the two proteins found to be downregulated in MND patient plasma derived EVs, relative to NC. Lastly, on the right column are listed proteins observed to be downregulated in ANI patients, relative to No NCI.

MND		ANI
Upregulated	Downregulated	Downregulated
PELP1	TLN2	ALB
FGG	DLGAP1	SERPINA1
HBB		IGHM
IGHV1-69-2		APOA1
FGB		GC
A2M		SLC12A3
HP		NNT
IGKV2D-28		TLN2
		DLGAP1
		IGHA1
		IGKV3-20
		IGLV1-47
		FGA
		APOC1
		LRIG1
		APCS
		IGHV3-73
		C3
		TF
		IGKC
		MYO7A
		UPB1
		ANKDD1A
		IGKV3D-7

## Data Availability

The datasets generated are not publicly available. However, the subset of data used in the study are available from the corresponding author upon request.
